# The South Carolina State Dental Association

**Published:** 1874-08

**Authors:** J. W. Norwood

**Affiliations:** Recording Secretary.


### ARTICLE VIII.

### *The South Carolina State Dental Association.*

This association held its annual session in the city of Charleston, on the 16th, 17th, 18th and 19th of June. The meeting was the most successful and interesting of any held since its organization, and in point of representation, the most numerous ; and resolutions were offered to have the proceedings published as soon as they could be compiled for the purpose. A bill was framed to be presented at the next session of the Legislature of the State, to regulate the practice of dentistry in South Carolina.

Many interesting papers were read and discussed, and some new ideas eliminated. The following officers were elected to serve for the ensuing year:

*President.*—Theodore F. Chupein, Charleston, S. C.
1*st Vice President.*—G. F. S. Wright, Pomaria, S. C.
2*nd Vice President.*—M. Bissel, Camden, S. C.
*Corresponding Secretary.*—C. C. Patrick, Charleston, S. C.
*Recording Secretary.*—J. W. Norwood, Greenville, S. C.
*Treasurer.*—T. W. Bonchier, Chirard, S. C.

J. W. Norwood, *Recording Secretary.*

